# Inhibitory Effect of Jinwujiangu Prescription on Peripheral Blood Osteoclasts in Patients with Rheumatoid Arthritis and the Relevant Molecular Mechanism

**DOI:** 10.1155/2023/4814412

**Published:** 2023-02-08

**Authors:** Ying Huang, Xueqin Gao, Yang An, Ping Zeng, Changming Chen, Wukai Ma, Xueming Yao

**Affiliations:** ^1^Guizhou University of Traditional Chinese Medicine, Guiyang, Guizhou Province 550025, China; ^2^The 2nd Hospital Affiliated with Guizhou University of Chinese Traditional Medicine, Guiyang, Guizhou Province 550003, China

## Abstract

Rheumatoid arthritis (RA) is a chronic progressive autoimmune disease characterized with high recurrence, high disability, poor prognosis, and long treatment cycles. Versus western medicine, traditional Chinese medicine has the traits of definite efficacy, low toxicity, and side effects in the treatment of RA. Moreover, traditional Chinese medicine also has the advantages of multiple targets, multiple links, and multiple approaches. This study was committed to exploring the effect of Jinwujiangu prescription on peripheral blood osteoclasts in those patients with RA and relevant molecular mechanisms. We first identified 159 common targets by online pharmacology, and there were correlations among these targets; besides, the main signaling pathways involved were inclusive TNF signaling pathway, rheumatoid arthritis, IL-17 signaling pathway, NF-kappa B signaling pathway, Toll-like receptor signaling pathway, etc. Through experimental verification, we found that PBMC cells extracted from human peripheral blood could be successfully induced into osteoclasts, and Jinwujiangu prescription inhibited the generation of osteoclasts from PBMCs of RA patients. CCK-8 and flow cytometry showed that osteoclast viability was significantly decreased and osteoclast apoptosis was significantly increased in the HIF-1*α* interference group; low-, medium-, and high-dose Jinwujiangu prescription groups; sinapine group; and hydroxychloroquine control group. Moreover, Jinwujiangu prescription and sinapine could inhibit the production of cytokines in peripheral blood osteoclasts and inhibit autophagy in RA patients. The expression level of mTOR was significantly increased in both Jinwu middle- and high-dose groups. In conclusion, this study demonstrated that sinapine, the active target in Jinwujiangu prescription, can act as a HIF-1*α* inhibitor; activate the mTOR pathway; downregulate the level of autophagy rate, ATG5, beclin-1, and LC3 expression; and inhibit the occurrence of autophagy. The trial registration number of the study is KYW2021010.

## 1. Introduction

Rheumatoid arthritis (RA), whose clinical symptoms are synovial inflammation and the progressive destruction of cartilage and bone tissue, is a common chronic autoimmune disease [1]. The epidemiological characteristics of RA are high incidence, high recurrence rate, and high disability rate, affecting 0.5%~1% of the global population [2]. RA onset within six months can lead to irreversible synovial injury and bone loss near the inflammatory joint, while patients will have subchondral bone destruction and systemic bone loss within two years and gradually develop into joint mobility limitation [3]. Osteoclasts are the main effector cells that play a role in the process of joint destruction [4]. It was found that a large number of activated osteoclasts and mature osteoclasts were found at the site of bone destruction in RA patients. Several proinflammatory cytokines associated with the pathogenesis of RA can increase osteoclast differentiation [5]. On these clues, one of the main goals of the current clinical treatment of RA is to regulate osteoclast generation.

Antirheumatic drugs, nonsteroidal anti-inflammatory drugs, and glucocorticoids are the main drugs in the treatment of RA. However, in fact, these drugs could only relieve the clinical symptoms, and they not only have limited efficacy but also have adverse reactions [6, 7]. Since the pathogenesis of RA is complex and single-target drugs are difficult to obtain good clinical efficacy, it is necessary to start with multicomponent and multitarget drugs in order to conquer RA [8].

The rapid development of bioinformatics in the past decade has made network pharmacology a research hotspot [9]. Network pharmacology is a discipline that constructs the component-target-disease network of drugs through online data retrieval and data analysis, which is able to demonstrate the interrelationship between drugs and diseases to clarify the specific mechanism of action [10]. This study intended to explore the possible main components and targets of Jinwujiangu prescription in the treatment of RA by network pharmacology method for cluster analysis and explore the effects of different concentrations of Jinwujiangu prescription on osteoclast differentiation and bone resorption induced from PBMCs of RA patients. We also discussed the role of osteoclasts in the bone destruction of RA and the possible molecular mechanism of Jinwujiangu prescription for the prevention and treatment of RA, so as to provide a new way and idea for the treatment of RA.

## 2. Materials and Methods

### 2.1. Network Pharmacological Analysis

In light of the main chemical constituents of Jinwujiangu prescription, we predicted the corresponding targets according to the principle of collateral pharmacology. The targets of all active ingredients were downloaded from the SwissTargetPrediction website (http://swisstargetprediction.ch/) [11]. Subsequently, we retrieved RA the keyword in OMIM database (https://www.omim.org), Gene Cards database (https://www.genecards.org), and TTD database (https://db.idrblab.net/ttd), then the species was set as human, and gene projects were selected to collect information about disease targets of RA; on this, we plotted the protein-protein interaction (PPI) network with an online platform STRING [12]. We constructed the medicine-ingredient-target-disease network graph by Cytoscape 3.6.0 software [13]. In addition, we analyzed the enrichment of GO and KEGG pathways of overlapping genes by Cytoscape plug-in ClueGO and finally obtained the enrichment results for analysis.

### 2.2. Source of Peripheral Blood and Preparation of Jinwujiangu Prescription

Peripheral blood of RA patients was provided by the Second Affiliated Hospital of Guizhou University of Traditional Chinese Medicine for this study. And this study was approved by the Medical Ethics Committee of the hospital, and all study subjects signed written informed consent. Jinwujiangu prescription has the ingredients of Cibotium barometz 15 g, Homalomena occulta 10 g, Periploca forrestii Schltr 10 g, Zaocys dhumnades 10 g, small blue vine 15 g, pseudoginseng 3 g, Radix Paeoniae Alba 15 g, turmeric 15 g, and liquorice 3 g. Jinwujiangu prescription (9 times the adult dosage, the adult dose of Jinwujiangu is 90 g) was put into the extraction jar, soaked in 500 mL of water for 30 min, decocted twice, combined with the medicine solution, concentrated to 1 : 2 (mL: g), cooled, kept in closed storage for 24~48 h, and filtered, and ethanol was recovered from the filtrate to obtain the refined solution. After that, the refined solution was sealed away from light and stored at 4°C for later use. The low, medium, and high concentrations of Jinwujiangu prescription were 0.06, 0.6, and 6.0 mg/mL, respectively.

### 2.3. PBMC Cell Isolation

The fresh anticoagulated whole blood was diluted with an equal volume of PBS. 15 mL of separation solution was added into the centrifuge tube, and then, another 15 mL of diluted blood was added into the tube to make the added blood spread above the separation liquid level and to ensure a clear boundary between the two fluids. The mixture was placed at room temperature and centrifuged at 1000 g for 30 min. After centrifugation, there will be a distinct stratification: the top layer was the diluted plasma layer, the middle was the transparent separation fluid layer, the white membrane layer between the plasma and separation fluid was the lymphocyte layer, and the centrifuge tube bottom was the red blood cells and granulocytes. The tunica albuginea cells were gently transferred to a 15 mL clean centrifuge tube and washed with 10 mL PBS. The above liquid was centrifuged at 250 g for 10 min, the supernatant was discarded, and the cells were resuspended in 5 mL PBS and centrifuged at 250 g for another 10 min. The previous procedure was repeated, and then, the complete medium was resuspended in PBS, and the cell concentration was adjusted to 1 × 10^5^ cells/mL and cultured in 24-well plates.

### 2.4. Differentiation Induction and Identification to Osteoclasts

24 h after PBMC cell suspension inoculation, the medium was changed to induction medium containing 100 ng/mL RANKL and 50 ng/mL M-CSF, and the solution was changed every two days for continuous culture for 14 days. 14 days after that, tartrate-resistant acid phosphatase (TRAP) staining was performed for identification.

### 2.5. Experimental Cell Grouping

The PBMC cell suspension was seeded and cultured in a 24-well culture plate, which was replaced with induction medium after 24 h, and cultured continuously for 14 days. The cells were divided into the blank group, rabbit serum control group, HIF-1*α* interference group, HIF-1*α* interference control group, Jinwujiangu prescription low-dose group, Jinwujiangu prescription medium-dose group, Jinwujiangu prescription high-dose group, sinapine group, and hydroxychloroquine control group. After 14 days of induction, the treatment of each group was finished at the same time, and the cells were collected for subsequent detection.

### 2.6. CCK-8

Groups of treated cells were taken from a 96-well plate, and 10 *μ*L of CCK-8 solution was added to each well (bubbles generated in wells must be avoided as they would affect OD readings). The plates were placed in the incubator for 4 h. The absorbance at 450 nm was measured with a microplate reader. At the same time, blank wells (medium, CCK) and control wells (cells, medium, CCK without any treatment) were set.

### 2.7. Flow Cytometry Detection

The cell suspension after group treatment was collected, mixed slightly, transferred to a centrifuge tube, centrifuged at 1000 RPM for 5 min, then the supernatant was discarded, the cells were collected, and the cells were gently resuspended with PBS and counted. A total of 1 × 10^5^ resuspended cells were centrifuged at 1000 RPM for 5 min, the supernatant was discarded, and the cells were gently resuspended by adding 195 *μ*L Annexin V-FITC-A binding solution. 5 *μ*L Annexin V-FITC-A was added to the liquid, gently mixed, and incubated at room temperature (25°C) for 10 min in the dark (it can also be wrapped in aluminum foil to avoid light). The mixture was centrifuged at 1000 RPM for 5 min, the supernatant was discarded, and cells were gently resuspended by adding 190 *μ*L Annexin V-FITC-A binding solution. Finally, 10 *μ*L PI staining solution was added to the mixture, gently mixed, and placed in an ice bath to avoid light; then, flow cytometry was performed.

### 2.8. ELISA

Specimen processing is as follows: cell culture supernatant was collected and centrifuged at 1000 × g for 20 min. After centrifugation, the supernatant was taken for detection, and the supernatant was stored either at -20°C or -80°C to avoid repeated freeze-thaw. Cells were treated with room temperature equilibration kit. The required cell culture plate was taken, and the standard wells and sample wells were set. The standard wells were added with different concentrations of standard 50 *μ*L; 50 *μ*L of the sample was added to the sample well; 100 *μ*L of horseradish catalase-labeled antibody was added to each well of the standard and sample, and the well plate was sealed with a plate sealing membrane and incubated at 37°C for 60 min. After incubation at room temperature, the liquid in plate wells was removed, and the remaining liquid was removed with absorbent paper. Then, each well was filled with the washing solution, then the washing solution was discarded after standing for 1 min, and the remaining liquid was removed with absorbent paper, which was repeated for 5 times. 50 *μ*L of substrates A and B was added to each well and incubated at 37°C for 15 min in the dark. At the end of incubation, 50 *μ*L of stop solution was added to each well, and OD values of each well were measured at a wavelength of 450 nm within 15 min. The reagents used in this section were shown as follows: Human HIF-1*α* Elisa Kit (Huamei, CSB-E12112h), Human CXCL8 Elisa Kit (Huamei, CSB-E04641h), Human CCL20 Elisa Kit (Huamei Biota, CSB-E04667h), Human TNF-*α* Elisa Kit (Huamei Biota, CSB-E09315h), Human IL-6 Elisa Kit (Huamei Biota, CSB-E04628h), Human IL-17 Elisa Kit (Huamei, CSB-E12819h), Human TRAP Elisa Kit (Huamei, CSB-E13415h), and Human IL-1 Elisa Kit (Apixin, Abs510002).

### 2.9. qPCR Detection

Total RNA was extracted with RNAiso Plus kit, and the obtained total RNA was transcribed with Goldenstar™ RT6 cDNA Synthesis Kit Ver.2 and the RNA Reverse Transcription Kit (Beijing, Qingke, TSK302M). The mRNA expression level was detected by real-time PCR. The quantification kit was 2x T5 Fast qPCR Mix (SYBR Green I) (Beijing, Qingke, TSE002). All the primer sequences used were shown as follows:

HIF-1*α*-FTCTTTTACCATGCCCCAGATTC

HIF-1*α*-RATTAGGCTCAGGTGAACTTTGTCTAGT

mTOR-FGCTTCTTCCGTTCCATCTCCT

mTOR-RGGGTCTGGGCGTATCAATTCT

LC3-FAAGCTTATGCCGTCGGAGAAGA

LC3-RGAATTCTTACACTGACAATTTCATCCC

ATG5-FCCTAGCCTCATACCCCCAG

ATG5-RCGTTGATCCCAGGAGTCACAA

Beclin-1-FTTGGCACAATCAATAACTTCAGGC

Beclin-1-RCCGTAAGGAACAAGTCGGTATCTC

TRAP-FGACCACCTTGGCAATGTCTCTG

TRAP-RTGGCTGAGGAAGTCATCTGAGTTG

CTSK-FCTCCTCCCTACCCTTCCTTCT

CTSK-RCTGTTGTCTGGCTTCGTTTCG

CTR-FGCAGGAAGATGTATGCTTTGA

CTR-RCTTTACAACAGCTAGGTCCTG

MMP9-FGAGATGCGTGGAGAGTCGAAA

MMP9-RTAGGTGATGTTGTGGTGGTGC

GAPDH-FCTGGGCTACACTGAGCACC

GAPDH-RAAGTGGTCGTTGAGGGCAATG

### 2.10. Western Blot

The total protein was extracted by RIPA lysate, and then, 500 *μ*g of total protein was taken from each sample and mixed with 5x SDS loading buffer in a ratio of 4 : 1. The protein concentration after mixing was about 3.3 *μ*g/*μ*L. The protein was denatured by heating in a metal bath at 100°C for 6 min. 60 *μ*g of each denatured total protein was used for loading. The concentrated glue was electrophoresed at 80 V, and then, the voltage was converted to 120 V, until bromophenol blue reached the bottom of the glue plate but just did not overflow. The current was adjusted to a constant current of 250 mA, and the transfer time was determined according to the molecular weight of the protein. After electrophoresis, the membrane was removed and the front and back sides of the membrane were marked. After blocking with 5% skim milk blocking solution for 1 h at room temperature, the primary antibody was diluted 1 : 1000 with primary antibody diluent and incubated at 4°C overnight. The secondary antibody was diluted to a certain concentration (1 : 2000) with blocking solution and then incubated for 1.0 h at room temperature. The ECL exposure solution was evenly mixed according to liquid A/liquid B = 1 : 1 and then evenly covered on the whole film. After 1 min of reaction, the ECL exposure solution was put into the exposure instrument for exposure detection. The antibodies used were as follows: HIF-1*α* (ABclonal, A11945), mTOR (ABclonal, A11355), LC3 (ABclonal, A19665), ATG5 (ABclonal, A11427), beclin-1(ABclonal, A17028), TRAP (ABclonal, A0962), CTSK (ABclonal, A1782), CTR (ABclonal, A15043), MMP9 (ABclonal, A0289), and GAPDH (ABclonal, A19056).

### 2.11. Flow MDC Single-Standard Detection

The subtreated cell suspension was collected, mixed slightly, transferred to a centrifuge tube, and centrifuged at 1000 RPM for 5 min. The supernatant was discarded, the cells were collected, and the cells were gently resuspended in PBS and counted. A total of 1 × 10^5^ resuspended cells were centrifuged at 1000 RPM for 5 min, then the supernatant was discarded, and 5 *μ*L anti-LC3/FITC (Bioss, China, BS-8878R-FITC) was added, and the cells were gently resuspended. The cells were incubated at room temperature (25°C) and shielded from light for 10 min. The cells could also be shielded from light by wrapping in aluminum foil. The cells were centrifuged at 1000 RPM for 5 min, then the supernatant was discarded, and the cells were gently resuspended with 200 *μ*L PBS and placed in an ice bath under light. After that, flow cytometry was performed.

### 2.12. Transmission Electron Microscopy Observation on Autophagy

The cell suspension after modeling treatment was collected and centrifuged in a 15 mL centrifuge tube at 1000 RPM for 5 min. The supernatant was discarded, and the fixed solution diluted 1 : 5 (3% glutaraldehyde: PBS buffer) was slowly added along the tube wall with a pipestraw, and the cells were resuspended and allowed to stand at 4°C for 5 min. After standing, the cell suspension was transferred to a 1.5 mL EP tube with the tip bottom and centrifuged at 12000 RPM for 10 min, and the supernatant was gently discarded to retain the precipitate. 1 mL pipette was used to slowly add 3% glutaraldehyde fixation solution along the tube wall (cells could not be blown away). If the solution could not be detected immediately after collection, it could be stored at 4°C environment. Fixation: samples were prefixed with 3% glutaraldehyde and refixed with 1% osmium tetroxide. Dehydration: pyruvate dehydration step by step and dehydrating agent concentration 30%⟶50%⟶70%⟶80%⟶90%⟶95%⟶100% (100% concentration for 3 times). Osmosis and embedding: the dehydrated sample was successively passed through the osmotic solution of dehydrating agent and epoxy resin (model Epon 812), with the ratio of 3 : 1, 1 : 1, and 1 : 3, respectively, for 30~60 min in each step. The permeated sample block was placed in the appropriate mold, coated with embedding solution, and then heated and polymerized to form a solid matrix (embedded block), which was prepared for the next step of sectioning. Ultrathin section: the ultrathin section with a thickness of about 50 nm is prepared by the ultrathin section mechanism, then floats on the liquid surface of the knife groove, and then dredges to the copper net. The sections were stained with uranyl acetate for 10-15 min, followed by lead citrate for 1-2 min, at room temperature, and observed under a JEM-1400 PLUS transmission electron microscope.

### 2.13. Statistical Analysis

The data obtained from three independent experiments were analyzed by GraphPad Prism 8.0.1, and the results were displayed in a manner of mean ± standard deviation (SD). The Tukey test was used for comparison between groups by one-way ANOVA. Parameters with *P* < 0.05 were considered significant differences.

## 3. Results

### 3.1. Target Identification through Network Pharmacological Method

**Figure 1:**
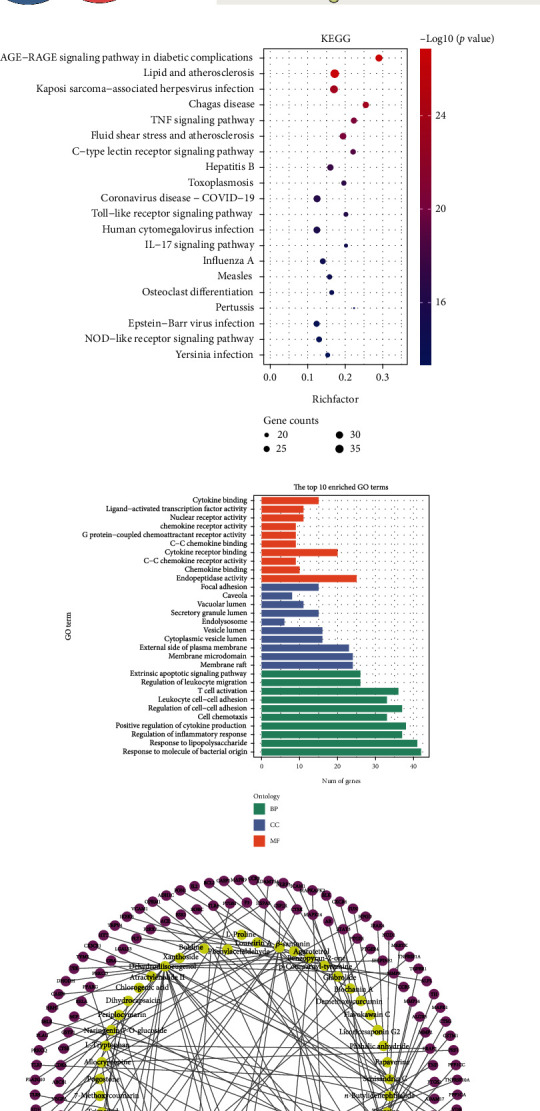
Target identification through network pharmacological method. (a) Venn diagram on rheumatoid arthritis-related genes and Jinwujiangu prescription targets. (b) PPI network diagram: interaction analysis of 159 target proteins was performed via STRING database. (c) Circle diagram of KEGG pathway enrichment. The right outer layer represented metabolic pathways, and the left represented genes. (d) GO analysis bars of biological processes, molecular functions, and cellular components. GO terms were on the left, and the abscissa represented gene counts. (e) Network diagram of compounds and targets.

A total of 1035 RA related genes were obtained from the database, and 1105 Jinwujiangu prescription targets were obtained from the TCMSP database; afterwards, 159 intersections of the two targets were obtained after standardization by UniProt database (Figure 1(a)). Interaction analysis to the proteins of the 159 targets was performed in STRING database, and the central nodes were TLR4, IL-1*β*, MMP9, VEGFA, etc. (Figure 1(b)). 159 targets were annotated with GO function and sorted by −log*P* value, and the top ten biological processes were regulation of inflammatory response, cell chemotaxis, positive regulation of cytokine production, and so on; cell components were concentrated in vesicle lumen, membrane microdomain, membrane raft, and so on; molecular function displays show that endopeptidase activity, chemokine binding, and cytokine receptor binding regulate RA (Figure 1(d)). KEGG enrichment analysis of common targets showed that the regulation effect of Jinwujiangu prescription on RA was related to TNF signaling pathway, rheumatoid arthritis, IL-17 signaling pathway, NF-kappa B signaling pathway, and Toll-like receptor signaling pathway (Figure 1(c)). The network map of Jinwujiangu prescription candidate compounds and RA targets was established by Cytoscape 3.6.0, and the active target sinapine was used for subsequent experimental analyses (Figure 1(e)).

### 3.2. Induction and Identification to Osteoclast

**Figure 2:**
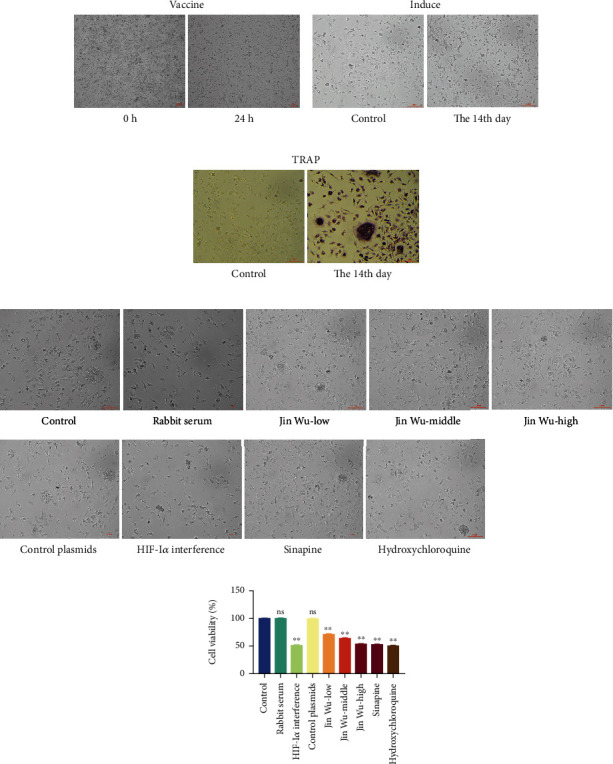
Induction and identification to osteoclasts. (a) Subsequent culture to PBMC cells. (b) PBMC cells were induced by M-CSF and RANKL. (c) TRAP was to stain the induced PBMC. (d) The osteoclast growths in each group. (e) Cell viability analysis on the cells in each group by CCK-8. A value whose ^∗∗^*P* < 0.01 indicated a significant difference when compared with the blank group. Note: scale bars = 100 *μ*m, ×200.

PBMC cells extracted from human peripheral blood showed small round shape and bright color after separation and lysis. After being cultured in the incubator for 24 h, more cells were observed adherent to the wall (Figure 2(a)). After 14 days of coinduction of M-CSF and RANKL, more multinucleated cells appeared, with larger cell bodies and stronger refractive ability than other cells, and the shape was like omelette or long strip (Figure 2(b)). After TRAP staining, a large number of multinucleated cells with purplish red granular precipitate in the cytoplasm were observed under the microscope, which were osteoclasts (Figure 2(c)).

### 3.3. Jinwujiangu Prescription Inhibited Osteoclast Generation from PBMCs in RA Patients

In this section, we identified that sinapine was a targeted inhibitor of HIF-1*α* through experiments and determined the effect of different doses of Jinwujiangu prescription on osteoclast generation by PBMC. The experimental animals were divided into 9 groups, blank group, rabbit serum control group, HIF-1*α* interference group, HIF-1*α* interference control group, Jinwujiangu prescription low-dose group, Jinwujiangu prescription medium-dose group, Jinwujiangu prescription high-dose group, sinapine group, and hydroxychloroquine control group, which were treated according to the groups during induction. The experiments showed that the interference of Jinwujiangu prescription, HIF-1*α*, and sinapine all led to a decrease in the number of osteoclasts generated by PBMCs (Figure 2(d)).

**Figure 3:**
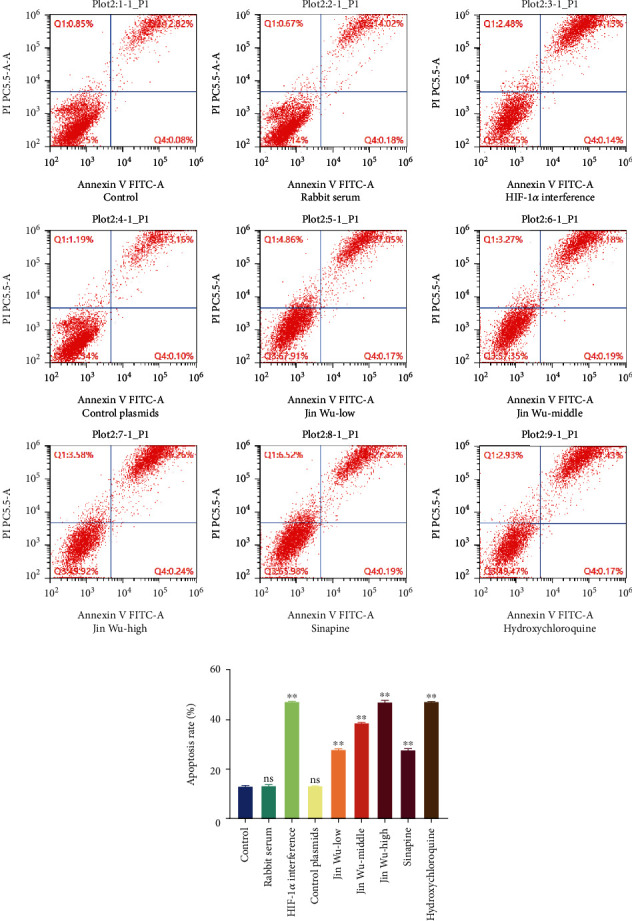
A comparison to the cell death rate in each group. (a) The flow cytometry detection results on cell apoptosis of each group. (b) The statistical analysis onto the apoptosis of each group. A value whose ^∗∗^*P* < 0.01 indicated a significant difference when compared with the blank group.

**Figure 4:**
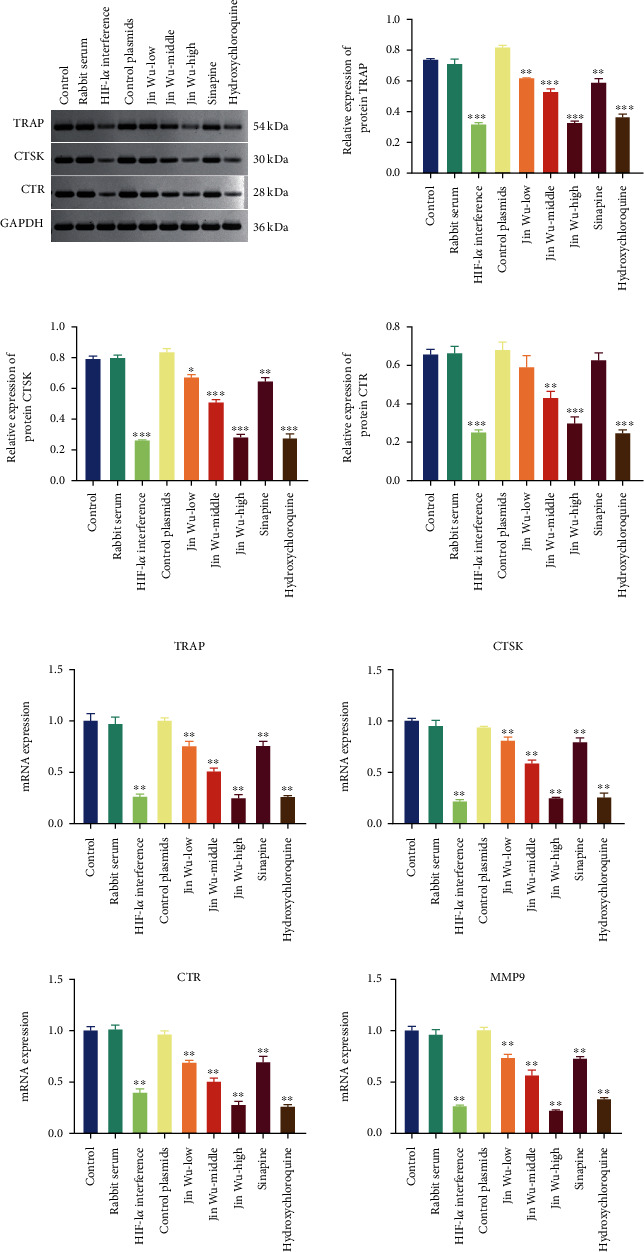
Bar chart on the relative expressions of osteoclast differentiation-related mRNAs and proteins. (a) Protein expression assays. (b) mRNA expression assays. Note: in comparison to the blank group, the value of ^∗^*P* < 0.05 represented a significant difference; the value of ^∗∗^*P* < 0.01 represented a highly significant difference.

The results of flow cytometry were consistent with the results of CCK-8 assay; when compared with control group, there was no significant difference in cell viability and apoptosis rate of rabbit serum group and HIF-1*α* interfered with control group; osteoclast viability was significantly decreased in the HIF-1*α* interference group; Jinwujiangu prescription group with low, medium, and high doses; sinapine group; and hydroxychloroquine control group (*P* < 0.05, Figure 2(e)), and osteoclast apoptosis was significantly increased (*P* < 0.05, Figure 3). The expression levels of osteoclast differentiation-related genes TRAP, CTSK, CTR, and MMP9 were significantly decreased in the HIF-1*α* interference group; Jinwujiangu prescription low-dose, medium-dose, and high-dose groups; and sinapine and hydroxychloroquine control groups (*P* < 0.05, Figure 4(b)). Western blot showed that the protein expressions of TRAP, CTSK, and CTR were basically consistent with the results of qPCR (Figure 4(a)).

### 3.4. Jinwujiangu Prescription Inhibited the Generation of Cytokine

**Figure 5:**
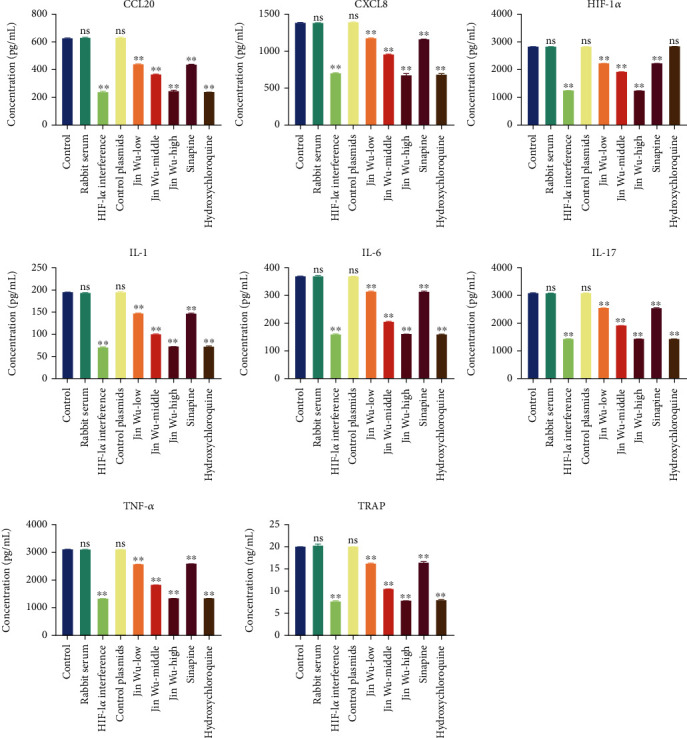
The ELISA detection on the contents of CCL20, CXCL8, HIF-1*α*, TNF-*α*, IL-1, IL-6, IL-17, and TRAP in each group of cells. A value whose ^∗∗^*P* < 0.01 indicated a significant difference when compared with the blank group.

Results of ELISA showed that when compared with the blank group, there were no significant differences in the levels of HIF-1*α*, CXCL8, CCL20, TNF-*α*, IL-1, IL-6, IL-17, and TRAP in the rabbit serum group and HIF-1*α* interference control group; contents of CXCL8, CCL20, TNF-*α*, IL-1, IL-6, and IL-17 secreted by the HIF-1*α* interference group, Jinwujiangu prescription low-dose group, Jinwujiangu prescription medium-dose group, Jinwujiangu prescription high-dose group, and sinapine groups were all decreased (Figure 5).

### 3.5. Jinwujiangu Prescription Inhibited Autophagy

**Figure 6:**
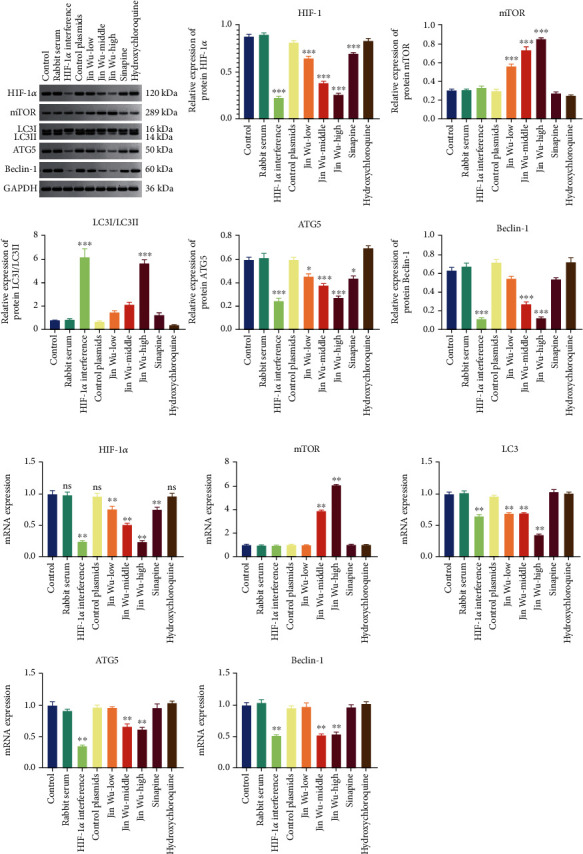
Bar chart on the relative expressions of autophagy-related mRNAs and proteins. (a) Protein expression assays. (b) mRNA expression assays. Note: in comparison to the blank group, the value of ^∗^*P* < 0.05 represented a significant difference; the value of ^∗∗^*P* < 0.01 represented a highly significant difference.

In this section, the effect of Jinwujiangu prescription on autophagy was verified, and the expression of autophagy-related genes was measured. In contrast to the control group, the expressions of HIF-1*α*, LC3, ATG5, and beclin-1 in the HIF-1*α* interference group and middle-dose and high-dose Jinwujiangu groups were significantly decreased (*P* < 0.05). Expressions of HIF-1*α* and LC3 in the Jinwujiangu prescription low-dose group were also significantly decreased (*P* < 0.05). Expression level of mTOR in the Jinwujiangu prescription group and high-dose group was significantly increased (*P* < 0.05), but there was no significant change in other groups. Western blot showed that the protein expressions of HIF-1*α* and ATG5 were basically consistent with the results of qPCR. However, expressions of mTOR in the Jinwujiangu prescription low-dose, medium-dose, and high-dose groups were significantly increased (*P* < 0.05). The expression of LC3I/LC3II in the HIF-1*α* interference group and Jinwujiangu prescription high-dose group was significantly increased (*P* < 0.05), but there was no significant difference in the other groups (Figure 6).

**Figure 7:**
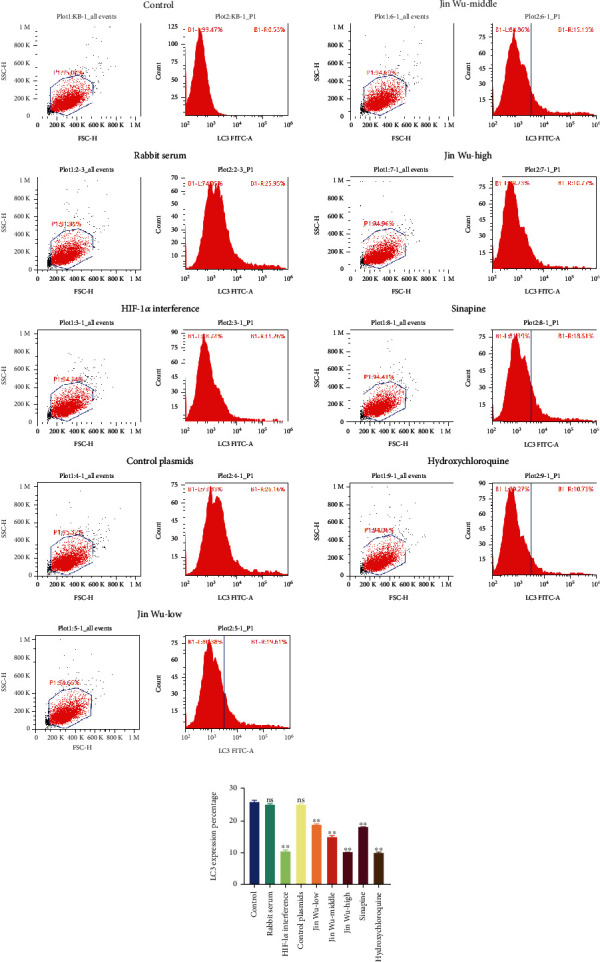
The percentage of MDC single standard (%) in each group. B1-L: negative areas represented the percentage of the cells without LC3 expression; B1-R: the positive areas represented the percentage of the cells expressing LC3. In comparison to the blank group, the value of ^∗^*P* < 0.05 indicated a significant difference; the value of ^∗∗^*P* < 0.01 indicated a highly significant difference.

**Figure 8:**
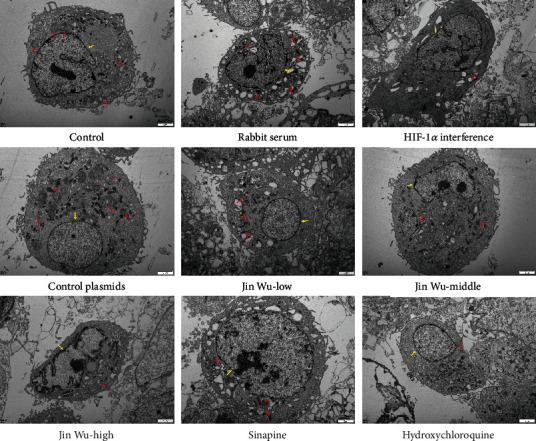
Transmission electron microscopy detection on the cells of each group. Yellow arrow indicated nucleus, and red arrow indicated autophagy. Scale bar = 2 *μ*m.

Subsequently, MDC was used to measure the expression of LC3 in each group, and the number of autophagosomes was observed via transmission electron microscopy. The results showed that when compared with the blank group, there was no significant difference in the expression of autophagy between the rabbit serum control group and HIF-1*α* interference control group; expressions of autophagy in the Hif-1*α* interference group, Jinwujiangu prescription low-dose group, Jinwujiangu prescription medium-dose group, Jinwujiangu prescription high-dose group, sinapine group, and hydroxychloroquine group decreased, which turned out significant differences (Figure 7). The number of autophagosomes in each group was observed by transmission electron microscopy. The results showed that there was no significant difference in the number of autophagosomes in the rabbit serum control group and HIF-1*α* interference control group compared with the blank group; the number of autophagosomes observed by transmission electron microscopy in the Hif-1*α* interference group, Jinwujiangu prescription low-dose group, Jinwujiangu prescription medium-dose group, Jinwujiangu prescription high-dose group, sinapine group, and hydroxychloroquine control group was relatively small (Figure 8).

## 4. Discussion

If early RA is not effectively treated, it will lead to irreversible joint damage, joint destruction, deformity, and even joint function loss and disability. Even after clinical remission, patients with advanced RA cannot recover their normal physiological function, which seriously reduces their working ability and quality of life. Jinwujiangu prescription has the functions of tonifying kidney and promoting blood circulation, dispelling wind and dehumidification, dredging collaterals, and relieving pain. With the purpose of fully elaborating the treatment mechanism of Jinwujiangu prescription for RA, this study analyzed the common targets of Jinwujiangu prescription and RA through network pharmacology, and 159 common targets were obtained. We also used PPI to analyze the interaction relationship between these targets, and TLR4, IL-1*β*, MMP9, and so on were in the central node. To fully describe the active matrix of Jinwujiangu prescription on RA, we performed GO functional annotation and KEGG enrichment analysis on 159 targets. Results of our experiments show that Jinwujiangu prescription's effect on RA was related to TNF signaling pathway, rheumatoid arthritis, IL-17 signaling pathway, NF-*κ*B signaling pathway, and Toll-like receptor signaling pathway.

TNF is a central cytokine in the pathophysiology of RA and a key cytokine in inflammation [14–17]. TNF reflects various effector functions associated with the pathogenesis of RA, which makes it proinflammatory at multiple levels [18]. In synovial cell cultures from RA patients, TNF blockade significantly reduces the production of other proinflammatory cytokines and chemokines, such as IL-1, IL-6, IL-8, or GM-CSF [19–21]. Results from related animal models of arthritis also confirmed the importance of TNF in RA [22]. Il-17 is produced spontaneously by the RA synovium, and increased levels of these IL-17 are associated with more severe joint injury and disease activity. Nf-*κ*b is considered by many studies to be an important downstream protein of multiple signaling pathways, which participates in the activation of multiple signaling pathways and plays an important role in immune inflammation and apoptosis. Reducing the phosphorylation of NF-*κ*B signaling pathway is one of the important ways to alleviate inflammation in RA [23]. TLR expression is increased in peripheral blood mononuclear cells, synovial tissue, synovial fluid, synovial macrophages, and synovial fibroblasts in patients with RA, and TLR2-9 may be involved in the pathogenesis of RA to different degrees. Studies have shown that blocking TLR2 prevents spontaneous cytokine release from RA in vitro synovial graft cultures [24], and TLR4 antagonists inhibit the spontaneous secretion of TNF-*α* and IL-1*β* from synovial tissue cultures in vivo [25]; all of this evidence implies that Jinwujiangu prescription may treat RA by acting on these signaling pathways.

In RA, a large number of osteoclasts accumulate in the synovial tissue, leading to resorption pits in the joints and local bone destruction [26]. Aberrant osteoclast production plays an important role in the development of RA, and this process is further actively regulated by proinflammatory cytokines such as TNF-*α* under pathogenic conditions [27]. To verify the effect of Jinwujiangu prescription on osteoclast generation, we first induced PBMC cells extracted from human peripheral blood by M-CSF and RANKL and confirmed that they could be induced into osteoclasts by TRAP staining. After HIF-1*α* interference, low, medium and high doses of Jinwujiangu prescription and sinapine were induced at the same time. The results showed that the number of osteoclasts generated was decreased. CCK-8 and flow cytometry showed the same results. The viability of osteoclasts in the HIF-1*α* interference group; low -, medium -, and high-dose Jinwujiangu prescription groups; and sinapine group were significantly decreased, and the apoptosis of osteoclasts was significantly increased. These results above suggested that Jinwujiangu prescription promotes the recovery of RA patients by reducing osteoclast formation.

Osteoclast differentiation requires a series of signaling pathways, transcription factors, and degrading enzymes to complete bone resorption. TRAP is one of the hallmark proteins of osteoclasts, which is highly expressed in both differentiated and mature osteoclasts, and plays an important role in bone resorption. CTSK, a member of cathepsin family, is a lysosomal protease, which is mainly expressed in osteoclasts [28]. Deletion of the CTSK gene in mouse hematopoietic cells or monocytes increased bone volume and bone formation rate, as well as the number of osteoclasts and osteoblasts [29]. MMP9, a member of the matrix metalloproteinase family, belongs to type IV collagenase and is essential for the degradation of bone organic matrix. This study showed that the expressions of osteoclast differentiation-related markers TRAP, CTSK, MMP9, and CTR were significantly reduced by the interference of Jinwujiangu, sinapine, and HIF-1*α*. These results indicated that sinapine, the active target of bone-enhancing activity of Jinwu, could act as an inhibitor of HIF-1*α* to inhibit the osteoclast differentiation of PBMC.

Studies have shown that cytokines still play a central regulatory role during the pathogenesis of RA [30]. There are also interactions within different cytokines during this course; for example, IL-17 increases the production of IL-1 and TNF by monocytes [31]. Previous studies have demonstrated the synergistic effects of IL-17, TNF, and IL-1 in many cell types such as synovial cells, chondrocytes, osteoblasts, or myoblasts [31]. Just as CCL20 promotes inflammation, the synergistic interaction between cellular inflammatory cytokines and their products would upregulate proinflammatory chemokines [32]. In addition, there are also a variety of key cytokines involved in the pathogenesis of RA exacerbating and maintaining inflammation This study found that HIF-1*α* interference and low, medium, and high doses of Jinwujiangu prescription and sinapine could reduce the contents of CXCL8, CCL20, TNF-*α*, IL-1, IL-6, and IL-17.

From the point of view of cell physiology and health, autophagy is an essential and conserved lysosomal degradation procession [33]. In addition, autophagy also regulates various cellular processes such as apoptosis, inflammation, pathogen clearance, and immune response, so it is regarded as a potential target for the treatment for many diseases [34]. Studies have shown that ATG5 is an essential inducible molecule for autophagy [35]. Beclin-1 is a protein molecule that plays a role in the initiation of autophagy, promoting the formation of autophagosomes by binding autophagosomes to lysosomes in bilayer membranes [36]. LC3 is a kind of autophagosome marker protein; of its subtypes, LC3I and LC3-II played a major role in the courses of autophagosome membrane elongation, membrane fusion, and transport [37, 38]. PI3K/AKT/M TOR is a signaling pathway associated with the initiation of autophagy. Activated mTOR might not only upregulate major cell cycle proteins and accelerate the cell cycle but also mediate cell growth and autophagy to participate in the occurrence and development of RA by inhibiting autophagy [39–41]. In this study, expressions of ATG5, LC3, and beclin-1 in cells were significantly decreased in the HIF-1*α* interference group and medium- and high-dose Jinwujiangu prescription groups. Expression of mTOR was significantly increased in the middle- and high-dose Jinwujiangu prescription groups. LC3I/LC3II expression was significantly increased in the HIF-1*α* interference group and high-dose Jinwujiangu prescription group. Through MDC detection and transmission electron microscope observation, we found that the expression of autophagosome LC3 was decreased in the bone building treatment group of Jinwujiangu prescription. In conclusion, this study proves that the active target sinapine in Jinwujiangu prescription can act as a HIF-1*α* inhibitor; activate the mTOR pathway; downregulate the level of autophagy rate, ATG5, beclin-1, and LC3 expressions; and inhibit the occurrence of autophagy.

## 5. Conclusion

We found that there were 159 targets of intersection within Jinwujiangu prescription and RA through network pharmacological analysis, and then, we carried out GO functional annotation and KEGG enrichment analysis onto these 159 targets. And it turned out that Jinwujiangu prescription on RA was highly associated with the signaling pathways of TNF, IL-17, NF-*κ*B, Toll-like receptor, and rheumatoid arthritis. In conclusion, we believed that Jinwujiangu prescription has inhibitory effects on osteoclast generation and cytokine production; besides, active monomer sinapine functions as an inhibitor of HIF-1*α*, and it is also able to inhibit osteoclast differentiation and autophagy.

## Data Availability

All data, models, and code generated or used during the study appear in the submitted article.

## Abbreviations

RA: Rheumatoid arthritis
PBMC: Peripheral blood mononuclear cells
HIF-1*α*: Hypoxia-inducible factor-1*α*
TRAP: Tartrate-resistant acid phosphatase
TNF-*α*: Tumor necrosis factor-*α*
IL-1: Interleukin-1
IL-6: Interleukin-6
IL-17: Interleukin-17
CTSK: Cathepsin K
CTR: Calcitonin receptor
ATG5: Autophagy-related proteins
LC3: Microtubule-associated protein 1A/1B-light chain 3
mTOR: Mammalian target of rapamycin.
